# Incidence and Susceptibility Pattern of Methicillin Resistant Coagulase-Negative Staphylococci Isolated From a Tertiary Care Hospital of Pakistan

**DOI:** 10.5812/jjm.8590

**Published:** 2014-01-01

**Authors:** Muhammad Usman Shah, Muhammad Farhan Akram, Javaid Usman, Fatima Kaleem

**Affiliations:** 1 Department of Microbiology, Army Medical College, National University of Sciences and Technology, Islamabad, Pakistan

**Keywords:** Antibiotics, Coagulase Negative Staphylococci, Methicillin Resistance

## Abstract

**Background:**

Methicillin resistant coagulase-negative Staphylococci are resistant organisms causing infections associated with high morbidity and mortality. Methicillin resistant *Staphylococcus epidermidis* (MRSE), is especially important with respect to admitted patients with indwelling catheters and other installed invasive devices where these organisms are known to be found. As a result, such lifesaving measures may prove fatal from subsequent infection and sepsis by these pathogens. Therefore, to limit such conditions in patients, the spread of MRSE and related organisms in the hospitals have to be effectively controlled.

**Objectives:**

This study was carried out to determine the frequency of methicillin resistant organisms among all isolated coagulase negative Staphylococci (CoNS) and to find effective antibiotics against these microorganisms.

**Patients and Methods:**

All samples sent to the lab were routinely processed according to standard microbiological procedures and the cultures yielding growth of CoNS were selected for the study. All samples containing CoNS collected over a 2 year-period, were included irrespective of patients' age and gender. The antibiogram of the organisms was recorded according to CLSI guidelines and the ratio of methicillin resistant organisms determined.

**Results:**

From a total of 299 isolated coagulase negative Staphylococci (CoNS), 40.1% were methicillin resistant. A high proportion of these organisms (more than 50%) were resistant to cephalosporins, aminoglycosides and quinolones while only a small number were found to show resistance to linezolid, minocycline, chloramphenicol and rifampicin. There were no resistant organisms against vancomycin.

**Conclusions:**

A considerable amount of methicillin resistant organisms found among CoNS in our region. The above stated antibiotics would prove effective in limiting these infections. Clinicians should keep these facts in mind while treating their patients.

## 1. Background

*Staphylococcus epidermidis* is the main organism among the coagulase negative Staphylococci (CoNS). It is a part of the normal flora of the skin but may act as a pathogen causing fatal infections which may have a significant incidence especially in the immune compromised patients (1). Likewise, it is also one of the most frequent organism causing post-operative surgical site and graft infections (2) as well as intravascular catheter and prostheses infection (3). The last mentioned infection is particularly significant as the increasing adoption of invasive procedures, prosthetic implants and percutaneous devices are used which are resulted in a high probability of subsequent infections. S. *epidermidis* and CoNS continue to be the major causes of sepsis (4) and meningitis in neonates and elderly hospitalized patients admitted for atopic dermatitis (2). 

The increasing number of resistant organisms causes difficulties to treat life threatening infections. This is a consequence of mass use of antibiotics (1) resulting in emergence of resistant genes giving rise to organisms like methicillin resistant *S*.* epidermidis* (MRSE). Therefore, with the reduced effectiveness of previous antibiotics and a rise of nosocomial infections, these resistant organisms resulted in increased morbidity and mortality rate of admitted patients into our hospitals (5). The resistance may be increased several folds with the production of enveloping biofilms. Such organisms are especially found in indwelling catheters and other instruments. These biofilm-associated catheter infections, responsible for recurrent CoNS infections in hospitalized premature neonates, are difficult to treat because of intrinsic resistance of biofilms to antibiotics (4). 

Currently, vancomycin is considered as the drug for eradicating such resistant organisms. However, cases of coagulase negative Staphylococci resistant to vancomycin are now appearing, as a majority that has developed the above mentioned biofilms (6). This makes these organisms extremely resistant to this antibiotic, resulting in therapeutic failure of the drug. Therefore, other drugs that have been recently introduced for such organisms, including linezolid, along with a few traditional ones such as minocycline, rifampicin and fusidic acid may need to be used which may prove to be more effective in treating such infections. Other means to overcome this resistance is the modification of the dosage regimens (e.g. using high-dose therapy), inhibiting the resistance mechanism (e.g. beta-lactamase inhibitors) or using an agent from a different class (7).

## 2. Objectives

Our study was aimed to determine the incidence of methicillin resistance among all of the coagulase negative Staphylococci isolated and to determine the effectiveness of various antibiotics to find out a suitable, yet cost effective treatment against these resistant organisms.

## 3. Patients and Methods

A descriptive, cross sectional study was carried out in the Microbiology department of Army Medical College, National University of Sciences and Technology, to determine the frequency and antibiogram of the methicillin resistance of all the CoNS isolated in the lab. This study was performed during 2 years, from January 2008 to January 2010. An informed consent form and institutional review board approval was received from the Army Medical College/ Military Hospital Pakistan review board. Informed consent was also taken from all the patients involved in the study. The studied samples were including blood, pus, urine, intravenous catheter tips, double lumen tip samples, catheter tip samples, umbilical swabs and wound swabs collected from both hospital admitted and outdoor patients.

Each sample was cultured onto blood agar plates and incubated at 37 °C for 24 hours. The characteristic isolates were aseptically isolated and identified as *Staphylococcus* by standard methods, including colonial morphology, Gram staining and catalase test. The isolates with coagulase and DNAase test positive results were considered as *S. aureus* and those with negative results were considered as CoNS. Final identification was done by API Staph kit and results were interpreted using API manufacturer manual instruction. Oxacillin (1 μg) and cefoxitin (30 μg) disks were used to assess the susceptibility of the isolates to methicillin. 

The isolates were considered as methicillin resistant if the zone of inhibition was < 25 mm for cefoxitin. Antibiotic susceptibility of the isolates as methicillin resistant was tested by Kirby-Bauer disc diffusion as recommended by the Clinical and Laboratory Standards Institute (1997). The following antibiotic disks (Oxoid-UK) were used: cephalaxin (30 μg), cephradine (30 μg), ciprofloxacin (5 μg), quinopristin/dalfopristin (15 μg), gentamicin (30 g), erythromycin (15 g), levofloxacin (5 μg), tetracycline (30 g), rifampicin (5 g), teicoplanin (30 g), minocycline (30 µg), chloramphenicol (30 µg), linezolid (30 µg) and vancomycin (30 µg). The isolates were inoculated on Mueller Hinton Agar (Oxoid-UK) containing 5% NaCl and incubated at 35 °C for 24 hours.

All isolates, irrespective of the age and sex of the patient, were included in the study and the cultures yielding growth of coagulase-negative Staphylococci were selected for the study and their antibiogram recorded according to CLSI guidelines. Data was entered in and analyzed using “Microsoft Excel 2007”. The selected strains in this study had the potential of being clinically significant on the basis of source and quantity and infective agent or both.

## 4. Results

**Figure 1.**
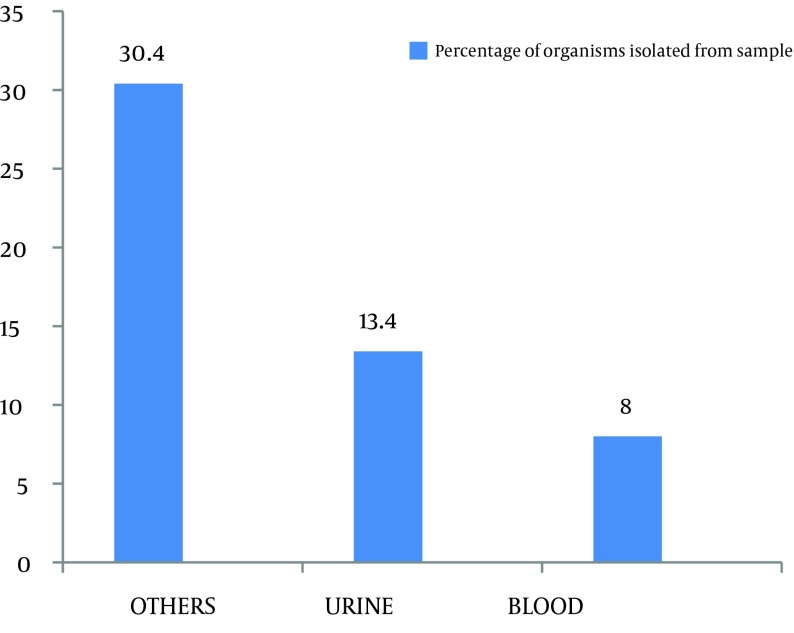
Frequency of Organisms Isolated From Various Samples

**Figure 2.**
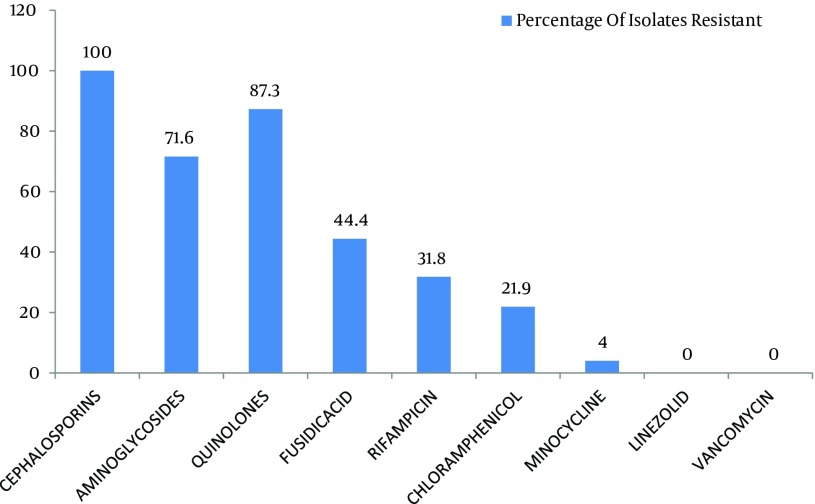
Antibiogram of Methicillin Resistant Coagulase Negative Staphylococci

Out of 299 coagulase negative Staphylococci isolates, 120 (40.1%) were found to be methicillin resistant which isolated from pus, urine, blood and other miscellaneous samples (Figure 1). The most effective antibiotics were vancomycin, linezolid, chloramphenicol, minocycline, and rifampicin. The efficacy of the first two was 100% followed by minocycline with only 4% effectiveness on isolated resistant organisms. Chloramphenicol and rifampicin was moderately effective with 21.9% and 31.8% resistance. Antibiotics that proved ineffective included fuscidic acid (44%), quinolones (87.3%) and aminoglycosides (71.6%) (Figure 2).

## 5. Discussion

Out of 299 isolates, 120 (40.1%) were found to be methicillin resistant coagulase negative Staphylococci (Figure 1). This amount was substantially higher than the 29.79% reported by Asma Bashir et al. and 16.2% by Issam Raad et al. (8 , 9). This difference may be explained by the different time periods in which the studies were carried out. The continuous and indiscriminate use of antibiotics during this time period resulted in an increase in the proportion of organisms with the resistant genes, hence, giving a higher incidence of resistant species. Unless this problem is managed in time, more and more organisms will become resistant. Such genes may even be transferred to other unrelated pathogens that are still sensitive to our current arsenal of antibiotics. Another study carried out by Issam Raad et al. in Texas University showed that more than 80% of nosocomial *S. epidermidis *isolates were methicillin-resistant (6). 

The five most effective drugs against the methicillin resistant coagulase negative Staphylococci were vancomycin, linezolid, chloramphenicol, minocycline, and rifampicin. None of the organisms were found to be resistant against linezolid. This is in accordance to a study by Sacar et al. showing that the antibiotic was highly effective in reducing the colony counts in MRSE-infected vascular Dacron grafts in rats which was comparable to the efficacy of vancomycin in the same study (10). Chlorampheniacol was moderately effective with 21.9% resistance (Figure 2). An earlier study by Fukada et al. showed that this drug had an efficacy rate of 81% against methicillin resistant *S *. *aureus *eye infections (2). 

It had a relatively better efficacy in such resistant organisms, compared to groups of antibiotics, which allow the administration of this antibiotic for treatment of infections caused by such organisms. Vancomycin was highly effective with 100% effectiveness, which was greater than 81.2% reported in previous studies (6). However, it has been reported that the failures in vancomycin treatments are possibly due to the biofilm synthesis, and rifampicin high efficacy, the combination of these antibiotics has been advised (11). This is in accordance with our study in which the mentioned drug was moderately effective against the resistant organisms with 31% resistance. 

This study also pointed out the possibility that vancomycin may be used in combination with linezolid, rifampicin or chloramphenicol, all of the latter drugs have acceptable efficacy against the above mentioned organisms. Linezolid may also be combined to rifampicin for greater efficacy and lower chances of resistance development, as shown by a study the additive effectiveness in a number of cases. Another antibiotic that should be highlighted is minocycline. Its efficacy against the resistant organisms was satisfactory. One study in particular concluded the its high efficiency in eradicating microorganisms embedded in fresh and mature biofilm adhering to catheter surfaces (6). This is important since this drug is much less expensive than both linezolid and vancomycin and has much better efficacy than the other tested antibiotics, that placed it on the top of first line drugs against the resistant organism. Other further studies provided evidences on the possibility of combining minocycline with rifampicin to coat vascular catheters to reduce the probability of infection (12).

Fusidic acid was effective in 55.6% of cases. This moderate degree of resistance was predicted by a study, reported that it was in-vitro active but the resistance developed if it was used as a single drug treatment and that greater results may be achieved if used in combination with other antibiotics such as rifampicin. This study also concluded that a high level of resistance was found among the organisms against other antibiotics including aminoglycosides and quinolones (Figure 2). Similar results were obtained by Yameen and coworkers for ciprofloxacin and cephalosporins (13). Such high resistivity patterns for the above mentioned drugs could be explained by previous practiced using single drug therapy as well as their excessive use in the hospitals and community resulting in decreased efficacy. 

Our study demonstrated that chloramphenicol, vancomycin, linezolid and minocycline all have excellent inhibitory effects against methicillin resistant coagulase negative Staphylococci (Figure 2). These may be used in infections including infective endocarditis, post operation surgical site infections, neonate meningitis and Staphylococcal eye infections, all of which caused by above mentioned organisms as one of the more important causative agents. The oral administration and affordable cost of minocycline, chloramphenicol and linezolid makes these drugs proper to be used for the treatment of such resistant infections. Furthermore, impregnation of medical devices by these agents would decrease the incidence of nosocomial infections which lessen the burden on our health care systems. This study can be further extended to include more samples, newly developed antibiotics and their combinations with other agents allowing us to effectively manage resistant CoNS, in both community and hospital. 
